# Crystal structure of an inorganic pyrophosphatase from *Chlamydia trachomatis* D/UW-3/Cx

**DOI:** 10.1107/S2053230X22002138

**Published:** 2022-02-28

**Authors:** Jasmine Maddy, Bart L. Staker, Sandhya Subramanian, Jan Abendroth, Thomas E. Edwards, Peter J. Myler, Kevin Hybiske, Oluwatoyin A. Asojo

**Affiliations:** a Department of Chemistry and Biochemistry, Hampton University, 100 East Queen Street, Hampton, VA 23668, USA; b Center for Global Infectious Disease Research, Seattle Children’s Research Institute, 307 Westlake Avenue North, Suite 500, Seattle, WA 98109, USA; c Seattle Structural Genomics Center for Infectious Disease (SSGCID), Seattle, Washington, USA; d Beryllium, 7869 NE Day Road West, Bainbridge Island, WA 98102, USA; e Departments of Pediatrics, Global Health, and Biomedical Informatics & Medical Education, University of Washington, Seattle, Washington, USA; f Division of Allergy and Infectious Diseases, Department of Medicine, University of Washington, Seattle, Washington, USA

**Keywords:** *Chlamydia trachomatis*, inorganic pyrophosphatase, infectious diseases, undergraduate education and training, Seattle Structural Genomics Center for Infectious Disease

## Abstract

*Chlamydia trachomatis* is the leading cause of bacterial sexually transmitted infections globally and is one of the most commonly reported infections in the United States. There is a need to develop new therapeutics due to drug resistance and the failure of current treatments to clear persistent infections. Structures of potential *C. trachomatis* rational drug-discovery targets, including *C. trachomatis* inorganic pyrophosphatase (*Ct*PPase), have been determined by the Seattle Structural Genomics Center for Infectious Disease. Inorganic pyrophosphatase hydrolyzes inorganic pyrophosphate during metabolism. Furthermore, bacterial inorganic pyrophosphatases have shown promise for therapeutic discovery. Here, a 2.2 Å resolution X-ray structure of *Ct*PPase is reported. The crystal structure of *Ct*PPase reveals shared structural features that may facilitate the repurposing of inhibitors identified for bacterial inorganic pyrophosphatases as starting points for new therapeutics for *C. trachomatis.*

## Abstract 2

*Chlamydia trachomatis* is the leading cause of bacterial sexually transmitted infections. *C. trachomatis* inorganic pyrophosphatase (CtPPase) hydrolyzes inorganic pyrophosphate during metabolism. A 2.2 Å resolution X-ray structure of CtPPase reveals shared structural features that may facilitate the repurposing of inhibitors identified for bacterial inorganic pyrophosphatases as starting points for new therapeutics.

## 1. Introduction

Chlamydiae are obligate intracellular bacteria that infect a wide range of eukaryotes, including humans, animals, insects and free-living amoebae. *Chlamydia trachomatis* is a Gram-negative coccus that causes a commonly known sexually transmitted infection often called chlamydia. Chronic chlamydia infection often leads to genital, ocular and respiratory disease (Lorenzini *et al.*, 2010 ▸). The *Chlamydia* genus is phylogenetically distant from other bacteria, and 30% of its proteins are referred to as hypothetical proteins (Barta *et al.*, 2013 ▸). Genital chlamydia is a major public health concern, with over 1.8 million cases reported to the US Centers for Disease Control and Prevention (CDC) in 2019. Furthermore, chlamydia is the most common bacterial sexually transmitted infection globally and is a leading cause of infertility (van Bergen *et al.*, 2021 ▸; Dombrowski, 2021 ▸). The CDC recommends treating chlamydia in adults and adolescents with 100 mg doxycycline orally twice a day for seven days. Alternatively, a single 1 g oral dose of azithromycin or 500 mg levofloxacin can be administered. However, reinfection is common with all antibiotics, and compliance is low for doxycycline (Centers for Disease Control and Prevention, 2021 ▸). Efforts to identify new treatment strategies for chlamydia at the Seattle Structural Genomics Center for Infectious Disease (SSGCID) include structural studies of *C. trachomatis* proteins as the first steps towards rational drug discovery. *C. trachomatis* inorganic pyrophosphatase (*Ct*PPase) was one of the investigated proteins because inorganic pyrophos­phatases from other bacteria have shown promise as potentially selective targets (Pang *et al.*, 2016 ▸; Lv *et al.*, 2014 ▸). The production, crystallization and high-resolution structure of *Ct*PPase are presented here.

## 2. Materials and methods

### 2.1. Macromolecule production

Cloning, expression and purification were conducted as part of the Seattle Structural Genomics Center for Infectious Disease (SSGCID) following standard protocols described previously (Bryan *et al.*, 2011 ▸; Choi *et al.*, 2011 ▸; Serbzhinskiy *et al.*, 2015 ▸). The full-length gene for inorganic pyrophosphatase from *C. trachomatis* (*Ct*PPase; UniProt O84777) encoding amino acids 1–209 was PCR-amplified from gDNA using the primers shown in Table 1 ▸. The gene was cloned into the ligation-independent cloning (LIC) expression vector pBG1861 encoding a noncleavable hexahistidine tag (Aslanidis & de Jong, 1990 ▸; Choi *et al.*, 2011 ▸). Plasmid DNA was transformed into chemically competent *Escherichia coli* BL21(DE3)R3 Rosetta cells. The plasmid containing hexahistidine-tagged *C. trachomatis* inorganic pyrophosphatase (His-*Ct*PPase) was expression-tested and 2 l of culture were grown using auto-induction medium (Studier, 2005 ▸). The expression clone ChtrB.01427.a.B1.GE42413 is available at https://www.ssgcid.org/available-materials/expression-clones/.

His-*Ct*PPase was purified in a two-step protocol consisting of an immobilized metal-affinity chromatography (IMAC) step and size-exclusion chromatography (SEC). All chromatography runs were performed on an ÄKTApurifier 10 (GE Healthcare) using automated IMAC and SEC programs according to previously described procedures (Bryan *et al.*, 2011 ▸). Thawed bacterial pellets were lysed by sonication in 200 ml buffer consisting of 25 m*M* HEPES pH 7.0, 500 m*M* NaCl, 5% glycerol, 0.5% CHAPS, 30 m*M* imidazole, 10 m*M* MgCl_2_, 1 m*M* TCEP, 250 µg ml^−1^ AEBSF, 0.025% azide. After sonication, the crude lysate was clarified with 20 µl (25 U µl^−1^) Benzonase and incubated while mixing at room temperature for 45 min. The lysate was then clarified by centrifugation at 10 000 rev min^−1^ for 1 h using a Sorvall centrifuge (Thermo Scientific). The clarified supernatant was then passed over an Ni–NTA HisTrap FF 5 ml column (GE Healthcare) which was pre-equilibrated with loading buffer consisting of 25 m*M* HEPES pH 7.0, 500 m*M* NaCl, 5% glycerol, 30 m*M* imidazole, 1 m*M* TCEP, 0.025% azide. The column was washed with 20 column volumes (CV) of loading buffer and was eluted with loading buffer plus 250 m*M* imidazole in a linear gradient over 7 CV. Peak fractions, as determined by the UV absorbance at 280 nm, were pooled and concentrated to 5 ml. A Superdex 75 SEC column (GE Healthcare) was equilibrated with running buffer consisting of 25 m*M* HEPES pH 7.0, 500 m*M* NaCl, 5% glycerol, 2 m*M* DTT, 0.025% azide. The peak fractions were collected and analyzed for *Ct*PPase using SDS–PAGE. The SEC peak fractions eluted as a single large peak at a molecular mass of ∼80 kDa, suggesting a trimeric enzyme. Peak fractions were pooled and concentrated to 62 mg ml^−1^ using an Amicon purification system (Millipore). Aliquots of 200 µl were flash-frozen in liquid nitrogen and stored at −80°C until use for crystallization.

### 2.2. Crystallization

Purified His-*Ct*PPase was screened for crystallization in 96-well sitting-drop plates against the JCSG+ HTS (Rigaku Reagents) and MCSG1 (Anatrace) crystal screens. Equal volumes of protein solution (0.4 µl) and precipitant solution were set up at 287 K against a 80 µl reservoir in sitting-drop vapor-diffusion format. 3 m*M* inorganic pyrophosphate was added to the protein solution before crystallization experiments. Crystals were obtained using high sodium chloride and polyethylene glycol 3350 conditions (Table 2 ▸). A crystal was cryoprotected by exchange into precipitant supplemented with 15%(*v*/*v*) ethylene glycol and vitrified directly in liquid nitrogen.

### 2.3. Data collection and processing

Data were collected at 100 K on beamline 21-ID-F at the Advanced Photon Source, Argonne National Laboratory (see Table 3 ▸). Diffraction data (Table 3 ▸) were integrated using *XDS* and were reduced using *XSCALE* (Kabsch, 2010 ▸). Raw X-ray diffraction images are available at the Integrated Resource for Reproducibility in Macromolecular Crystallography at https://www.proteindiffraction.org.

### 2.4. Structure solution and refinement

The structure was solved by molecular replacement with *Phaser* (McCoy *et al.*, 2007 ▸) from the *CCP*4 suite of programs (Collaborative Computational Project, Number 4, 1994 ▸; Krissinel *et al.*, 2004 ▸; Winn *et al.*, 2011 ▸) using PDB entry 5ls0 (Grzechowiak *et al.*, 2019 ▸) as the search model. The structure was refined using iterative cycles of *Phenix* (Liebschner *et al.*, 2019 ▸) followed by manual rebuilding of the structure using *Coot* (Emsley & Cowtan, 2004 ▸; Emsley *et al.*, 2010 ▸). The quality of the structure was checked using *MolProbity* (Williams *et al.*, 2018 ▸). All data-reduction and refinement statistics are shown in Table 4 ▸. The structure was refined to a resolution of 2.25 Å. Coordinates and structure factors have been deposited in the Protein Data Bank (https://www.rcsb.org) with accession code 6we5.

## 3. Results and discussion

**Figure 1:**
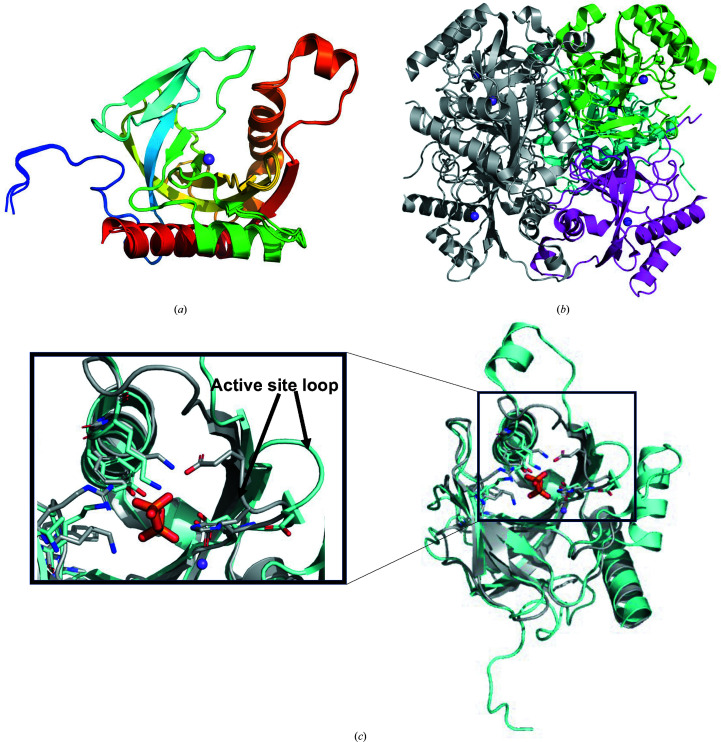
*Ct*PPase structure. (*a*) The superposed *Ct*PPase monomers are almost identical, with r.m.s.d.s of ∼0.3 Å for all atoms and ∼0.17 Å for C^α^ atoms. The monomers are colored from blue (N-terminus) to red (C-terminus). (*b*) A prototypical family I PPase hexamer was generated from the asymmetric unit trimer (monomers colored green, cyan and magenta) and a symmetry mate (shown in gray). The sodium ion bound in the active site of each monomer is shown as a purple sphere. (*c*) The *Ct*PPase active-site loop (cyan) is in the open conformation compared with the closed conformation of *M. tuberculosis* PPase (*Mt*PPase). The pyrophosphate (orange sticks) in the active site is from *Mt*PPase (PDB entry 5kde), while the sodium ion (purple sphere) is from *Ct*PPase (PDB entry 6we5).

*Ct*PPase is a small β-strand protein containing a core five-stranded oligonucleotide/oligosaccharide-binding (OB) fold. *Ct*PPase has the prototypical family I pyrophosphatase (PPase) topology. Family 1 PPases are ubiquitous in all kingdoms of life (Kajander *et al.*, 2013 ▸). The overall topology of *Ct*PPase resembles an open fist (or baseball mitt) with the substrate-binding cavity sitting in the palm, while β-strands form finger-like structures surrounding the active site (Fig. 1 ▸ *a*).

The *Ct*PPase structure was refined to 2.25 Å resolution in space group *C*222_1_ with three molecules in the asymmetric unit. Surface-area calculations by *PISA* (Krissinel, 2015 ▸) suggest a hexamer as the most likely biological assembly (Fig. 1 ▸ *b*). Hexamers were previously observed as the biological assembly in other well studied family I PPases, notably *E. coli* PPases (Cooperman *et al.*, 1992 ▸). The *Ct*PPase hexamer is similar to those of the well studied family I PPases. Electron density modeled as an Na atom was observed in the active site of each monomer. The active site is where the hydrolysis of pyrophosphate into two phosphate ions occurs. Despite the addition of pyrophosphate to the crystallization buffer, no density was observed for pyrophosphate or phosphate ions. Additionally, the flexible active-site loop is in the open conformation indicative of an apo structure without any substrate or product in the active site of *Ct*PPase (Fig. 1 ▸ *c*). Future studies will include investigating whether the presence of the N-terminal hexahistidine tag renders *Ct*PPase inactive and unable to hydrolyze pyrophosphate or form the biological hexamer in solution, or whether additional ions or cofactors need to be added to the enzyme before crystallization to generate the structure of the complex with pyrophosphate or phosphate.

**Figure 2:**
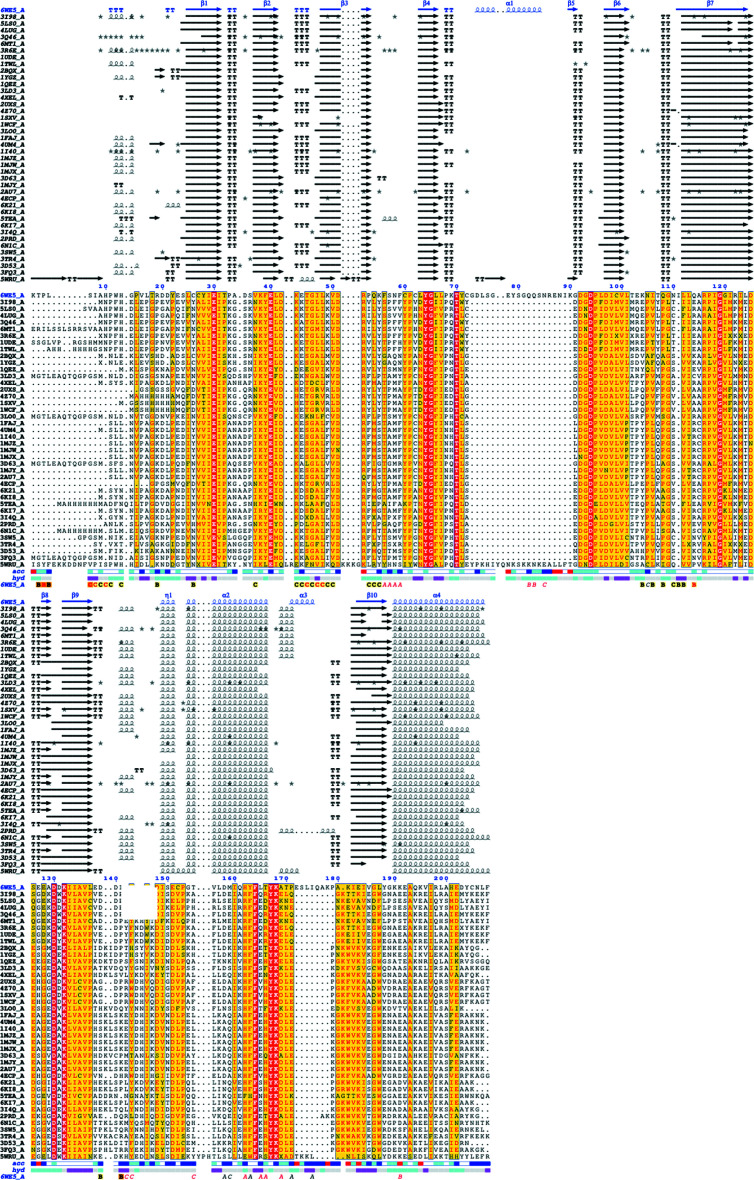
An *ENDscript* alignment identifies conserved residues in *Ct*PPase and PPases. Multi-sequence alignment of *Ct*PPase with 41 closest PPases obtained by a *BLAST* search against the PDBAA database. Identical and conserved residues are highlighted in red and yellow, respectively. Alternate residues are highlighted with gray stars. The different secondary-structure elements shown are α-helices (α), 3_10_-helices (η), β-strands (β) and β-turns (TT).

Since bacterial inorganic pyrophosphatases have shown promise as potentially selective targets (Pang *et al.*, 2016 ▸; Lv *et al.*, 2014 ▸), *Ct*PPase was compared with other structures to determine whether it could be a viable drug target. *PDBeFold* analysis (http://www.ebi.ac.uk/msd-srv/ssm/; Krissinel & Henrick, 2004 ▸), the *DALI *server (http://ekhidna2.biocenter.helsinki.fi/dali/; Holm, 2020 ▸) and *ENDscript* analysis (Gouet *et al.*, 2003 ▸; Robert & Gouet, 2014 ▸) were used to identify the closest structural neighbors of *Ct*PPase. These analyses revealed that despite <37% sequence similarity, *Ct*PPase shares significant secondary-structural similarity with several family I PPases, including some that have shown promise as drug targets (see supporting information and Fig. 2 ▸). The supporting information includes detailed results of the *DALI* (Supplementary Fig. S1) and *PDBeFold* (Supplementary Table S1) analyses. The overall core structure of *Ct*PPase is highly similar to other bacterial PPases except for two major insertions (residues 71–86 and residues 170–180; Figs. 2 ▸ and 3 ▸). These insertions are on the exterior surface of the hexamer and do not participate in the formation of the hexamer or interact with the active site (Fig. 1 ▸ *c*).

**Figure 3:**
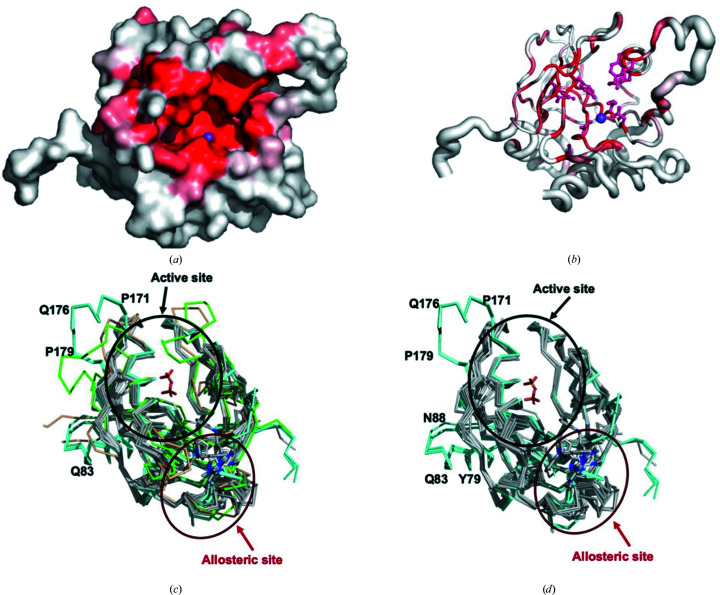
Structural comparison of *Ct*PPase with other PPases. (*a*) Solvent-accessible surface area colored by sequence conservation. Residues clustered in the active-site cleft are identified by the sodium ion present in the crystal structure of *Ct*PPase (magenta sphere). (*b*) Coil diagram calculated by *ENDscript*. The circumference of the ribbon (sausage) represents the relative structural conservation compared with 41 other PPase structures (the same structures as indicated in Fig. 2 ▸). Thinner ribbons represent more conserved regions, while thicker ribbons represent less conserved regions. The ten identical residues cluster within or in proximity to the active site. Identical residues are indicated by red regions on the surface and a red ball-and-stick representation in ribbon diagrams. The sodium ion bound in the active site of each monomer is shown as a purple sphere. (*c*) Comparison of the active and allosteric sites of CtPPase (cyan) with bacterial PPases (gray) and eukaryotic PPases (*Homo sapiens* PPase, PDB entry 7btn, green; *Plasmodium falciparum* PPase, PDB entry 5wru, brown). (*d*) The same view of the structures without the eukaryotic PPases. The top ten unique bacterial PPases were selected from the *ENDscript *alignment. All three monomer chains of *Ct*PPase are shown.

A comparison of *Ct*PPase with 41 other PPases deposited in the Protein Data Bank using *ENDscript* identified 19 identical residues which cluster in the active-site pocket (Figs. 2 ▸ and 3 ▸). The active-site region contains a D-(S/G/N)-D-P-ali-D-ali-ali motif, where ali is C/I/L/M/V (Kankare *et al.*, 1994 ▸). *PDBeFold* analysis also revealed that, as expected, bacterial PPases were structurally most similar to *Ct*PPase (Supplementary Table S1). The most similar structure was from *Thermococcus thioreducens*, followed by *Acinetobacter baumannii*, with root-mean-square differences (r.m.s.d.s) of 1.18 and 1.33 Å over 196 and 162 residues, respectively. The top PPases that showed structural similarity are listed in Supplementary Table S1. Our preliminary analysis revealed structural differences between bacterial and eukaryotic PPases that may possibly be exploited for inhibitor design (Fig. 3 ▸ *c*).

A manual search of the entire PDB for structures of PPases from other organisms identified 80 different ligand-bound PPase structures. The majority of ligands were metals, ions, substrate or substrate mimics. Most ligands were bound in the active site. However, there were four structures with ligands bound outside the active site: two structures of *Mycobacterium tuberculosis* PPase (*Mt*PPase), PDB entries 5kde and 5kd7 (Pang *et al.*, 2016 ▸), and two structures of *Burkholderia pseudo­mallei* PPase (*Bp*PPase), PDB entries 3ej2 and 3ej0 (Van Voorhis *et al.*, 2009 ▸). The *Mt*PPase ligands are low-micromolar IC_50_ allosteric inhibitors (Pang *et al.*, 2016 ▸). The *Bp*PPase ligands were discovered from fragment-based screens at the Seattle Structural Genomics Center for Infectious Disease. Superposition of the *C. trachomatis* (*Ct*PPase) structures with *Mt*PPase and *Bp*PPase revealed that the ligands are small organic compounds that are located in a surface binding pocket on the opposite side to the pyrophos­phate binding pocket (Fig. 3 ▸ *c*).

**Figure 4:**
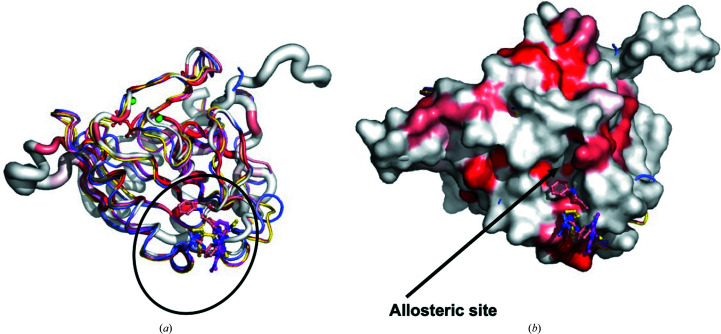
The bacterial PPase allosteric binding site. The putative allosteric binding site of *Ct*PPase identified from superposition of *Ct*PPase (PDB entry 6we5) with *Mt*PPase (PDB entries 5kde and 5kdf) and *Bp*PPase (PDB entries 3ej0 and 3ej2). (*a*) Coil diagram of *Ct*PPase (red and white) superimposed on the *Mt*PPase structures with allosteric inhibitors (PDB entries 5kde, yellow, and 5kdf, magenta) and *Bp*PPase bound with fragment compounds (PDB entries 3ej0, wheat, and 3ej2, blue). The location of the compounds is indicated with a black oval. The circumference of the coil represents the relative structural conservation compared with 41 other PPase structures (the same structures as indicated in Fig. 2 ▸). (*b*) A solvent-accessible surface diagram of *Ct*PPase calculated with *ENDscript* reveals a potential binding pocket labeled the allosteric site on the *Ct*PPase surface in proximity to the compounds.

The two previous studies on *Mt*PPase and *Bp*PPase suggest the possibility of an allosteric binding site that small-molecule inhibitors of bacterial PPases could target. Comparison of the *Ct*PPase structure with those of *Mt*PPase and *Bp*PPase shows that the loop adjacent to the putative allosteric binding site has moved into the pocket compared with *Mt*PPase and *Bp*PPase, closing off this site. Inspection of the solvent-accessible surface of *Ct*PPase reveals a medium-sized cleft that partially occupies the fragment-binding site of *Bp*PPase (Fig. 4 ▸). Future fragment-based screening targeting this cleft may generate allosteric inhibitors of *Ct*PPase.

## 4. Conclusion

We have determined the structure of an inorganic pyrophos­phatase (PPase) from *C. trachomatis*. The overall structure is a prototypical bacterial PPase with additional amino acids inserted beyond the conserved active site. *Ct*PPase has a pocket in proximity to the previously identified bacterial allosteric binding sites, suggesting the possibility of developing allosteric inhibitors of *Ct*PPase. While the preliminary structural studies are promising, future studies include validating the enzymatic activity of *Ct*PPase and probing the active and allosteric sites of *Ct*PPase with substrates and potential inhibitors.

## Supplementary Material

PDB reference: inorganic pyrophosphatase from *Chlamydia trachomatis* D/UW-3/Cx, 6we5

Supplementary Figure and Table. DOI: 10.1107/S2053230X22002138/tb5175sup1.pdf

## Figures and Tables

**Table 1:** Macromolecule-production information

Source organism	*Chlamydia trachomatis* (strain D/UW-3/Cx)
DNA source	Dr Kevin Hybiske (University of Washington, USA)
Forward primer	5′-CTCACCACCACCACCACCATATGTCTAAAACACCATTATCCATAGC-3′
Reverse primer	5′-ATCCTATCTTACTCACTTACATAAAAAGATTGCAATAGTCTTCGT-3′
Expression vector	pBG1861
Expression host	*E. coli* BL21(DE3)R3 Rosetta cells
Complete amino-acid sequence of the construct produced	MAHHHHHHMSKTPLSIAHPWHGPVLTRDDYESLCCYIEITPADSVKFELDKETGILKVDRPQKFSNFCPCLYGLLPKTYCGDLSGEYSGQQSNRENIKGDGDPLDICVLTEKNITQGNILLQARPIGGIRILDSEEADDKIIAVLEDDLVYGNIEDISECPGTVLDMIQHYFLTYKATPESLIQAKPAKIEIVGLYGKKEAQKVIRLAHEDYCNLFM

**Table 2:** Crystallization

Method	Vapor diffusion, sitting drop
Plate type	96-well Compact 300, Rigaku
Temperature (K)	287
Protein concentration (mg ml^−1^)	31
Buffer composition of protein solution	3 m*M* inorganic pyrophosphate, 25 m*M* HEPES pH 7.0, 500 m*M* NaCl, 5% glycerol, 2 m*M* DTT, 0.025% azide
Composition of reservoir solution	2 *M* NaCl, 0.1 *M* Tris pH 8.5, 25%(*v*/*v*) PEG 3350
Volume and ratio of drop	0.4 µl protein plus 0.4 µl reservoir
Volume of reservoir (µl)	80
Composition of cryoprotectant solution	2 *M* NaCl, 0.1 *M* Tris pH 8.5, 25%(*v*/*v*) PEG 3350, 15%(*v*/*v*) ethylene glycol

**Table 3:** Data collection and processing Values in parentheses are for the outer shell.

Diffraction source	Beamline 21-ID-F, APS
Wavelength (Å)	0.97872
Temperature (K)	100
Detector	RayoniX MX300HE CCD
Crystal-to-detector distance (mm)	260
Rotation range per image (°)	1
Total rotation range (°)	200
Space group	*C*222_1_
*a*, *b*, *c* (Å)	77.16, 121.19, 124.50
α, β, γ (°)	90, 90, 90
Mosaicity (°)	0.24
Resolution range (Å)	44.99–2.25 (2.31–2.25)
Total No. of reflections	226034 (16920)
No. of unique reflections	27864 (2010)
Completeness (%)	99.3 (99.2)
Multiplicity	8.1 (8.4)
〈*I*/σ(*I*)〉	25.24 (3.14)
*R*_r.i.m._ †	0.046 (0.661)
Overall *B* factor from Wilson plot (Å^2^)	56.57

† Estimated *R*_r.i.m._ = *R*_merge_[*N*/(*N* − 1)]^1/2^, where *N* is the data multiplicity.

**Table 4:** Structure refinement Values in parentheses are for the outer shell.

Resolution range (Å)	44.99–2.25 (2.31–2.25)
Completeness (%)	99.2
σ Cutoff	*F* > 1.34σ(*F*)
No. of reflections, working set	27834 (1787)
No. of reflections, test set	2028 (156)
Final *R*_cryst_	0.181 (0.275)
Final *R*_free_	0.228 (0.374)
No. of non-H atoms	
Protein	4694
Ion	3
Ligand	0
Water	65
Total	4762
R.m.s. deviations	
Bond lengths (Å)	0.004
Angles (°)	0.683
Average *B* factors (Å^2^)	
Protein	61.8
Ion	69.5
Ligand	0.0
Water	53.0
Ramachandran plot	
Most favored (%)	98.04
Allowed (%)	1.96
