# Supplementary material for: Crystal structure of an inorganic pyrophosphatase from Chlamydia trachomatis D/UW-3/Cx
Source: Acta Crystallogr F Struct Biol Commun. 2022 Feb 28;78(Pt 3):135–42. doi: 10.1107/S2053230X22002138 (PMC8900733; doi:10.1107/S2053230X22002138)
Supplement: Supplementary file 1 [file f-78-00135-sup1.pdf]

Acta Cryst F

STRUCTURAL BIOLOGY COMMUNICATIONS

Volume 78 (2022)

Supporting information for article:

Crystal structure of an inorganic pyrophosphatase from *Chlamydia trachomatis* D/UW-3/Cx

Jasmine Maddy, Bart L. Staker, Sandhya Subramanian, Jan Abendroth, Thomas E. Edwards, Peter J. Myler, Kevin Hybiske and Oluwatoyin A. Asojo

Figure S1 DALI analysis of *Ct*PPase (http://ekhidna2.biocenter.helsinki.fi/dali/) (Holm, 2020)

Table S1 Results from *PDBeFold P* (http://www.ebi.ac.uk/msd-srv/ssm/) analysis of *Ct*PPase (Krissinel & Henrick, 2004)

References

Holm, L. (2020). *Protein Sci* **29**, 128-140.

Krissinel, E. & Henrick, K. (2004). *Acta crystallographica. Section D, Biological crystallography* **60**, 2256-2268.

Dali alignment: 6we5

Each neighbour is shown in the pairwise Dali-alignment to 6we5A. Inserted segments relative to the top structure are hidden. You can check the 'Expand gaps' option in the summary page to see the complete sequence of the matched proteins. Uppercase means structurally equivalent positions with 6we5A. Lowercase means insertions relative to 6we5A. The first part shows the amino acid sequences of the selected neighbours. The second part shows the secondary structure assignments by DSSP (H/h: helix, E/e: strand, L/l: coil). The most frequent amino acid type is coloured in each column.

Show Stacked Sequence Logos

```
                      :         :         :         :         |         :         :         :         :       100         :         :         :         :         |         :         :      
0001 6we5A   KTPLSIAHPWHGPVLTRDDYESLCCYIEITPADSVKFELDKETGILKVDRPQKFSNFCPCLYGLLPKTYCGDLSGEYSGQQSNRENIKGDGDPLDICVLTEKNITQGNILLQARPIGGIRILDSEEADDKIIAVLEDDLVYGNIEDISECPGTVLDMIQHYFLTYKATPESLIQAK
0002 3q4wA   ------MNPFHELEPGPEVPEVVYALIEIPKGSRNKYELDKKTGLLKLDRVLYSPFFYPVDYGIIPQTWY------------------DDGDPFDIMVIM-REPVYPLTIIEARPIGIMKMEDSGDKDWKVLAVPVEDPYFNDWKDISDVPKAFLDEIAHFFQRYKEL--------
0003 5ucqC   ------MNPFHELEPGPEVPEVVYALIEIPKGSRNKYELDKKTGLLKLDRVLYSPFFYPVDYGIIPQTWY------------------DDGDPFDIMVIM-REPVYPLTIIEARPIGIMKMEDSGDKDWKVLAVPVEDPYFNDWKDISDVPKAFLDEIAHFFQRYKEL--------
0004 3q5vB   ------MNPFHELEPGPEVPEVVYALIEIPKGSRNKYELDKKTGLLKLDRVLYSPFFYPVDYGIIPQTWY------------------DDGDPFDIMVIM-REPVYPLTIIEARPIGIMKMEDSGDKDWKVLAVPVEDPYFNDWKDISDVPKAFLDEIAHFFQRYKEL--------
0005 3r5vC   ------MNPFHELEPGPEVPEVVYALIEIPKGSRNKYELDKKTGLLKLDRVLYSPFFYPVDYGIIPQTWY------------------DDGDPFDIMVIM-REPVYPLTIIEARPIGIMKMEDSGDKDWKVLAVPVEDPYFNDWKDISDVPKAFLDEIAHFFQRYKEL--------
0006 6mt2B   ---SVAAHPWHDLEIGPGAPNIFNCVVEITKGSKVKYELDKKTGMIKVDRILYSSVVYPHNYGFIPRTLC------------------EDNDPLDVLVLMQEPV-LPGCFLRARAIGLMPMIDQGEKDDKIIAVCADDPEYKHYTDFKELQPHRLMEIKRFFEDYKKN--------
0007 5ls0B   ---SVAAHPWHDLEIGPGAPQIFNVVVEITKGSKVKYELDKKTGLIKVDRILYSSVVYPHNYGFVPRTLC------------------EDNDPIDVLVIMQEPV-LPGCFLRARAIGLMPMIDQGEKDDKIIAVCVDDPEYKHYTDIKELPPHRLSEIRRFFEDYKKN--------
0008 3q3lC   ------MNPFHELEPGPEVPEVVYALIEIPKGSRNKYELDKKTGLLKLDRVLYSPFFYPVDYGIIPQTWY------------------DDGDPFDIMVIM-REPVYPLTIIEARPIGIMKMEDSGDKDWKVLAVPVEDPYFNDWKDISDVPKAFLDEIAHFFQRYKEL--------
0009 5ty5B   ------MNPFHELEPGPEVPEVVYALIEIPKGSRNKYELDKKTGLLKLDRVLYSPFFYPVDYGIIPQTWY------------------DDGDPFDIMVIM-REPVYPLTIIEARPIGIMKMEDSGDKDWKVLAVPVEDPYFNDWKDISDVPKAFLDEIAHFFQRYKEL--------
0010 4lugA   ------AHPWHDLEIGPGAPQIFNVVVEITKGSKVKYELDKKTGLIKVDRILYSSVVYPHNYGFVPRTLC------------------EDNDPIDVLVIMQEPV-LPGCFLRARAIGLMPMIDQGEKDDKIIAVCVDDPEYKHYTDIKELPPHRLSEIRRFFEDYKKN--------
0011 3i98F   ------MNPFHELEPGPEVPEVVYALIEIPKGSRNKYELDKKTGLLKLDRVLYSPFFYPVDYGIIPQTWY------------------DDGDPFDIMVIM-REPVYPLTIIEARPIGIMKMEDSGDKDWKVLAVPVEDPYFNDWKDISDVPKAFLDEIAHFFQRYKEL--------
0012 3r6eA   ------XNPFHELEPGPEVPEVVYALIEIPKGSRNKYELDKKTGLLKLDRVLYSPFFYPVDYGIIPQTWY------------------DDGDPFDIXVIX-REPVYPLTIIEARPIGIXKXEDSGDKDWKVLAVPVEDPYFNDWKDISDVPKAFLDEIAHFFQRYKEL--------
0013 6mt1A   ---SVAAHPWHDLEIGPGAPNIFNCVVEITKGSKVKYELDKKTGMIKVDRILYSSVVYPHNYGFIPRTLCE------------------DNDPLDVLVLMQE-PVLPGCFLRARAIGLMPMIDQGEKDDKIIAVCADDPEYKHYTDFKELQPHRLMEIKRFFEDYKKN--------
0014 3r6eC   ------XNPFHELEPGPEVPEVVYALIEIPKGSRNKYELDKKTGLLKLDRVLYSPFFYPVDYGIIPQTWY------------------DDGDPFDIXVIX-REPVYPLTIIEARPIGIXKXEDSGDKDWKVLAVPVEDPYFNDWKDISDVPKAFLDEIAHFFQRYKEL--------
0015 1udeA   ---------FHDLEPGPNVPEVVYALIEIPKGSRNKYELDKETGLLKLDRVLYTPFHYPVDYGIIPRTWY------------------EDGDPFDIMVIM-REPTYPLTIIEARPIGLFKMIDSGDKDYKVLAVPVEDPYFKDWKDISDVPKAFLDEIAHFFKRYKEL--------
0016 2au8A   -------SLLN-VPAGKDLPEDIYVVIEIPANADIKYEIDKESGALFVDRFMSTAMFYPCNYGYINHTLS------------------LDGDPVDVLVPT-PYPLQPGSVTRCRPVGVLKMTDEAGEDAKLVAVPHSSKEYDHIKDVNDLPELLKAQIAHFFEHYKDL-------K
0017 1obwB   -------SLLN-VPAGKDLPEDIYVVIEIPANADIKYEIDKESGALFVDRFMSTAMFYPCNYGYINHTLS------------------LDGDPVDVLVPT-PYPLQPGSVTRCRPVGVLKMTDEAGEDAKLVAVPHSSKEYDHIKDVNDLPELLKAQIAHFFEHYKDL-------K
0018 1i6tA   -------SLLN-VPAGKDLPEDIYVVIEIPANADIKYEIDKESGALFVDRFMSTAMFYPCNYGYINHTLS------------------LDGDPVDVLVPT-PYPLQPGSVTRCRPVGVLKMTDEAGEDAKLVAVPHSSKEYDHIKDVNDLPELLKAQIAHFFEHYKDL-------K
0019 2au7A   -------SLLN-VPAGKDLPEDIYVVIEIPANADIKYEIDKESGALFVDQFMSTAMFYPCNYGYINHTLS------------------LDGDPVDVLVPT-PYPLQPGSVTRCRPVGVLKMTDEAGEDAKLVAVPHSSKEYDHIKDVNDLPELLKAQIAHFFEHYKDL-------K
0020 2auuA   -------SLLN-VPAGKDLPEDIYVVIEIPNADPIKYEIDKESGALFVDRFMSTAMFYPCNYGYINHTLS------------------LDGDPVDVLVPT-PYPLQPGSVTRCRPVGVLKMTDEAGEDAKLVAVPHSSKEYDHIKDVNDLPELLKAQIAHFFEHYKDL--------
0021 1obwA   -------SLLN-VPAGKDLPEDIYVVIEIPANADIKYEIDKESGALFVDRFMSTAMFYPCNYGYINHTLS------------------LDGDPVDVLVPT-PYPLQPGSVTRCRPVGVLKMTDEAGEDAKLVAVPHSSKEYDHIKDVNDLPELLKAQIAHFFEHYKDL-------K
0022 5c5vA   TGRERQVSPWHDIPLYVR--NRYNFICEIPKWTRAKFEIATGENPIKQDRFYKH-GDMMWNYGAFPQTWESTE-VLFEA------GVTGDNDPVDAVEIGMT-QFKVGQVSAVKVLGVLGMIDEGKMDWKVVCISHNDPICRFMKDIHDVPPGCLDAIREWFRVYKIC-------G
0023 2prdA   ------ANLKS-LPVGDKAPEVVHMVIEVPRGSGNKYEYDPDLGAIKLDRVLPGAQFYPGDYGFIPSTLAE------------------DGDPLDGLVLST-YPLLPGVVVEVRVVGLLLMEDEKGGDAKVIGVVAEDQRLDHIQDIGDVPEGVKQEIQHFFETYKAEAKK-----
0024 2au6A   -------SLLN-VPAGKDLPEDIYVVIEIPANADIKYEIDKESGALFVDRFMSTAMFYPCNYGYINHTLS------------------LDGDPVDVLVPT-PYPLQPGSVTRCRPVGVLKMTDEAGEDAKLVAVPHSSKEYDHIKDVNDLPELLKAQIAHFFEHYKDL-------K
0025 1inoA   -------SLLN-VPAGKDLPEDIYVVIEIPANADIKYEIDKESGALFVDRFMSTAMFYPCNYGYINHTLS------------------LDGDPVDVLVPT-PYPLQPGSVIRCRPVGVLKMTDEAGEDAKLVAVPHSSKEYDHIKDVNDLPELLKAQIAHFFEHYKDL-------K
0026 5wrtB   -KSGERLSPWHDIPLFPNG-LLFNMVVEIPKNTRRKMEMQLRLTPIMQDREYA--STLYWNYGAFPQTWEDPR--EPGGREVFH--ARGDGDPLDVVEIGSEVL-PVGGVVPVKVLGALAMIDGGELDWKVLAIREGDPLFSQLNSVADVCRGVVPGIREWFRWYKLP--------
0027 1mjzA   -------SLLN-VPAGKDLPEDIYVVIEIPANADIKYEIDKESGALFVDRFMSTAMFYPCNYGYINHTLS------------------LDGDPVDVLVPT-PYPLQPGSVIRCRPVGVLKMTNEAGEDAKLVAVPHSSKEYDHIKDVNDLPELLKAQIAHFFEHYKDL-------K
0028 4um4C   -------SLLN-VPAGKDLPEDIYVVIEIPAADPIKYEIDKESGALFVDRFMSTAMFYPCNYGYINHTLS------------------LDGDPVDVLVPT-PYPLQPGSVIRCRPVGVLKMTDEAGEDAKLVAVPHSSKEYDHIKDVNDLPELLKAQIAHFFEHYKDL-------K
0029 2au9A   ------SLLN--VPAGKDLPEDIYVVIEIPNADPIKYEIDKESGALFVDRFMSTAMFYPCNYGYINHTLS------------------LDGDPVDVLVPT-PYPLQPGSVTRCRPVGVLKMTDEAGEDAKLVAVPHSSKEYDHIKDVNDLPELLKAQIAHFFEHYKDL--------
0030 6mt2D   ------AHPWHDLEIGPGAPNIFNCVVEITKGSKVKYELDKKTGMIKVDRILYSSVVYPHNYGFIPRTLC------------------EDNDPLDVLVLMQ-EPVLPGCFLRARAIGLMPMIDQGEKDDKIIAVCADDPEYKHYTDFKELQPHRLMEIKRFFEDYKK---------
0031 5wrtA   -KSGERLSPWHDIPLPNGR-LLFNMVVEIPKNTRRKMEMQLRLTPIMQDREYA--STLYWNYGAFPQTWEDP---EPGGREVFH--ARGDGDPLDVVEIGSEVL-PVGGVVPVKVLGALAMIDGGELDWKVLAIREGD-PLFSLNSVADVCRGVVPGIREWFRWYKLP--------
0032 1mjwA   -------SLLN-VPAGKDLPEDIYVVIEIPANADIKYEIDKESGALFVNRFMSTAMFYPCNYGYINHTLS------------------LDGDPVDVLVPT-PYPLQPGSVIRCRPVGVLKMTDEAGEDAKLVAVPHSSKEYDHIKDVNDLPELLKAQIAHFFEHYKDL-------K
0033 5cuyA   TGRERQVSPWHDIPLYVR--NRYNFICEIPKWTRAKFEIATGENPIKQDRFYKH-GDMMWNYGAFPQTWESTE--VLFE-----AGVTGDNDPVDAVEIGMT-QFKVGQVSAVKVLGVLGMIDEGEMDWKVVCISHSDPICHFLRDIHDVPPGCLDAIREWFRVYKICQGG-----
0034 6n1cA   ----------MEIQSGRDVPNEVNVIIEIPHGEPVKYEVDKKTGALFVDRFMTTAMFYPTNYGYIPNTLSE------------------DGDPVDVLVITPVPL-ISGAVISCRAVGMLKMTDESGVDAKILAVPTTSKMYQSMQTYQDIPQHLLLSIEHFFKHYKDL-------E
0035 5h4fA   -------SLLN-VPAGKDLPEDIYVVIEIPANADIKYEIDKESGALFVDRFMSTAMFYPCNYGYINHTLS------------------LDGDPVDVLVPT-PYPLQPGSVTRCRPVGVLKMTDEAGEDAKLVAVPHSSKEYDHIKDVNDLPELLKAQIAHFFEHYKDL-------K
0036 5teaD   -------DFNQILTPGD-DGGIINVVNEIPAGSNHKIEWNRKLAAFQLDRIEPAIFAKPTNYGFIPQTLD------------------EDGDELDVLLVTE-QPLATGVFLEARVIGVMKFVDDGEVDDKIVCVPADDRNGNAYKTLSDLPQQLIKQIEFHFNHYKDLK-------
0037 6n1cE   ----------MEIQSGRDVPNEVNVIIEIPHGEPVKYEVDKKTGALFVDRFMTTAMFYPTNYGYIPNTLSE------------------DGDPVDVLVITPVPL-ISGAVISCRAVGMLKMTDESGVDAKILAVPTTSKMYQSMQTYQDIPQHLLLSIEHFFKHYKDL-------E
0038 1mjwB   -------SLLN-VPAGKDLPEDIYVVIEIPANADIKYEIDKESGALFVNRFMSTAMFYPCNYGYINHTLS------------------LDGDPVDVLVPT-PYPLQPGSVIRCRPVGVLKMTDEAGEDAKLVAVPHSSKEYDHIKDVNDLPELLKAQIAHFFEHYKDL-------K
0039 6k27C   ---------YNNIPAGKDAPNDIYVIIEIPNAAPIKYEIDKDSDALFVDRFMGTAMFYPANYGYVPNTLS------------------EDGDPLDVLVVTP-YPVAAGSVIRCRPVGKLNMEDDGGIDAKLIAVPHESPLYKDVKEYTDLPQLLINQVEHFFSHYKDL-------P
0040 1udeB   ----------HDLEPGPNVPEVVYALIEIPKGSRNKYELDKETGLLKLDRVLYTPFHYPVDYGIIPRTWYED------------------GDPFDIMVIM-REPTYPLTIIEARPIGLFKMIDSGDKDYKVLAVPVEDPYFKDWKDISDVPKAFLDEIAHFFKRYKEL--------
0041 3eiyA   -------SFSN-VPAGKDLPQDFNVIIEIPQSEPVKYEADKALGLLVVDRFIGTGMRYPVNYGFIPQTLS------------------GDGDPVDVLVITPFPL-LAGSVVRARALGMLKMTDESGVDAKLVAVPHDCPMTANLKSIDDVPAYLKDQIKHFFEQYKAL-------K
0042 3ld3A   -----------DIGSGSNAPEEVNVVIEVSDSHPVKYEFDEKNGALWVDRFLPTAMYYPCNYGFIPNTIA------------------GDGDPVDVLVLA-RFPVMPGAVICVRPVGVLMMNDEKGEDAKVLAVPATDQYYGNIVNYSDLPSSFLDSISHFFSFYKKL-------K
0043 3d53D   --------FIKKIKAKANN-NEINVIIEIPNSGPIKYEFDKESGALFVDRFMQTTMSYPCNYGFIPDTLSN------------------DGDPVDVLVVAH-HPVVPGSVIKCRAIGVLMMEDESGLDEKIIAVPTSDITFDHIKELDDLCEMLKKRIVHFFEHYKDL-------K
0044 5cuvB   TGKELQVSPWHDIPLYVR--NRYNFICEIPKWTRAKFEIATGENPIKQDRFYKH-GDXXWNYGALPQTWESTD--VVFE-----GGYVGDNDPIDAIEIGXT-QFKVGQVGAVKVLGILGXIDDGQXDWKVICISHNDPICRFLKDIHDVPPGCLDAIHEWFRVYKICQ------G
0045 4z70C   -------------------AMQFDVTIEIPKGQRNKYEVDHETGRVRLDRYLYTPMAYPTDYGFIEDTLG------------------DDGDPLDALVLL-PQPVFPGVLVAARPVGMFRMVDEHGGDDKVLCVPAGDPRWDHVQDIGDVPAFELDAIKHFFVHYKDL-------P
0046 3emjA   ------------IKAKANN-NEINVIIEIPMSGPIKYEFDKESGALFVDRFMQTTMSYPCNYGFIPDTLSN------------------DGDPVDVLVVAH-HPVVPGSVIKCRAIGVLMMEDESGLDEKIIAVPTSDITFDHIKELDDLCEMLKKRIVHFFEHYKDL-------K
                      :         :         :         :         |         :         :         :         :       100         :         :         :         :         |         :         :      
0001 6we5A   LLLLLLLLLLLLLLLLLLLLLLEEEEEEELLLLLEEEEELLLLLLEEEEEELLLLLLLLLEEEELLLLLLLHHHHHHHHHHHLLLLLEELLLLLEEEEELLLLLLLLLEEEEEEEEEEEEELLLEELLEEEEEELLLLLLLLLLLHHHLLHHHHHHHHHHHHHHLLLLHHHHHLLL
0002 3q4wA   ------LLHHHHLLLLLLLLLLEEEEEEELLLLLEEEEELLLLLLEEEEEELLLLLLLLLEEEELLLLLL------------------LLLLLLEEEELL-LLLLLLLLEEEEEEEEEEEEELLEELLEEEEEELLLHHHLLLLLHHHLLHHHHHHHHHHHHHHLLHH--------
0003 5ucqC   ------LLHHHHLLLLLLLLLLEEEEEEELLLLLEEEEELLLLLLEEEEEELLLLLLLLLEEEELLLLLL------------------LLLLLLEEEELL-LLLLLLLEEEEEEEEEEEEEELLEELLEEEEEELLLHHHLLLLLHHHLLHHHHHHHHHHHHHHLLLL--------
0004 3q5vB   ------LLHHHHLLLLLLLLLLEEEEEEELLLLLEEEEELLLLLLEEEEEELLLLLLLLLEEEELLLLLL------------------LLLLLLEEEELL-LLLLLLLLEEEEEEEEEEEEELLEELLEEEEEELLLHHHLLLLLHHHLLHHHHHHHHHHHHHHLLHH--------
0005 3r5vC   ------LLHHHHLLLLLLLLLLEEEEEEELLLLLEEEEELLLLLLEEEEEELLLLLLLLLEEEELLLLLL------------------LLLLLLEEEELL-LLLLLLLEEEEEEEEEEEEEELLEELLEEEEEELLLHHHLLLLLHHHLLHHHHHHHHHHHHHHLLHH--------
0006 6mt2B   ---LLLLLHHHHLLLLLLLLLEEEEEEEELLLLLEEEEELLLLLLEEEEEELLLLLLLLLEEEELLLLLL------------------LLLLLLEEEEELLLLL-LLLLEEEEEEEEELEEELLEELLEEEEEELLLLLHHHLLLHHHLLHHHHHHHHHHHHHHLLHH--------
0007 5ls0B   ---LLLLLLLLLLLLLLLLLLEEEEEEEELLLLLEEEEELLLLLLEEEEEELLLLLLLLLEEEELLLLLL------------------LLLLLLEEEEELLLLL-LLLLEEEEEEEEELEEELLEELLEEEEEELLLLLLLLLLLHHHLLHHHHHHHHHHHHHHLLHH--------
0008 3q3lC   ------LLHHHHLLLLLLLLLLEEEEEEELLLLLEEEEELLLLLLEEEEEELLLLLLLLLEEEELLLLLL------------------LLLLLLEEEELL-LLLLLLLLEEEEEEEEEEEEELLEELLEEEEEELLLHHHLLLLLHHHLLHHHHHHHHHHHHHHLLHH--------
0009 5ty5B   ------LLHHHHLLLLLLLLLLEEEEEEELLLLLEEEEELLLLLLEEEEEELLLLLLLLLEEEELLLLLL------------------LLLLLLEEEELL-LLLLLLLEEEEEEEEEEEEEELLEELLEEEEEELLLHHHLLLLLHHHLLHHHHHHHHHHHHHHLLLL--------
0010 4lugA   ------LLLLLLLLLLLLLLLEEEEEEEELLLLLEEEEELLLLLLEEEEEELLLLLLLLLEEEELLLLLL------------------LLLLLLEEEEELLLLL-LLLLEEEEEEEEELEEELLEELLEEEEEELLLLLLLLLLLHHHLLHHHHHHHHHHHHHHLLHH--------
0011 3i98F   ------LLHHHHLLLLLLLLLLEEEEEEELLLLLEEEEELLLLLLEEEEEELLLLLLLLLEEEELLLLLL------------------LLLLLLEEEELL-LLLLLLLLEEEEEEEEEEEEELLEELLEEEEEELLLHHHLLLLLHHHLLHHHHHHHHHHHHHHLLHH--------
0012 3r6eA   ------LLHHHHLLLLLLLLLLEEEEEEELLLLLEEEEELLLLLLEEEEEELLLLLLLLLEEEELLLLLL------------------LLLLLLEEEELL-LLLLLLLLEEEEEEEEEEEEELLEELLEEEEEELLLHHHLLLLLHHHLLHHHHHHHHHHHHHHLLHH--------
0013 6mt1A   ---LLLLLLLLLLLLLLLLLLEEEEEEEELLLLLEEEEELLLLLLEEEEEELLLLLLLLLEEEELLLLLLL------------------LLLLLEEEEELLL-LLLLLLEEEEEEEEELEEELLEELLEEEEEELLLLLLLLLLLHHHLLHHHHHHHHHHHHHHLLHH--------
0014 3r6eC   ------LLHHHHLLLLLLLLLLEEEEEEELLLLLEEEEELLLLLLEEEEEELLLLLLLLLEEEELLLLLL------------------LLLLLLEEEELL-LLLLLLLLEEEEEEEEEEEEELLEELLEEEEEELLLHHHLLLLLHHHLLHHHHHHHHHHHHHHLLHH--------
0015 1udeA   ---------LLLLLLLLLLLLLEEEEEEELLLLLEEEEELLLLLLEEEEEELLLLLLLLLEEEELLLLLL------------------LLLLLLEEEELL-LLLLLLLLEEEEEEEEEEEEELLEELLEEEEEELLLHHHLLLLLHHHLLHHHHHHHHHHHHHHLLHH--------
0016 2au8A   -------LHHH-LLLLLLLLLLEEEEEEELLLLLEEEEELLLLLLEEEEEELLLLLLLLLEEEELLLLLL------------------LLLLLLEEEELL-LLLLLLLLEEEEEEEEEEEEELLEELLEEEEEELLLLLLLLLLLHHHLLHHHHHHHHHHHHHHLLLL-------L
0017 1obwB   -------LHHH-LLLLLLLLLLEEEEEEELLLLLEEEEELLLLLLEEEEEELLLLLLLLLEEEELLLLLL------------------LLLLLLEEEELL-LLLLLLLLEEEEEEEEEEEEELLEELLEEEEEELLLLLLLLLLLHHHLLHHHHHHHHHHHHHHLLLL-------L
0018 1i6tA   -------LHHH-LLLLLLLLLLEEEEEEELLLLLEEEEELLLLLLEEEEEELLLLLLLLLEEEELLLLLL------------------LLLLLLEEEELL-LLLLLLLLEEEEEEEEEEEEELLEELLEEEEEELLLLLLLLLLLHHHLLHHHHHHHHHHHHHHLLLL-------L
0019 2au7A   -------LHHH-LLLLLLLLLLEEEEEEELLLLLEEEEELLLLLLEEEEEELLLLLLLLLEEEELLLLLL------------------LLLLLLEEEELL-LLLLLLLLEEEEEEEEEEEEELLEELLEEEEEELLLLLLLLLLLHHHLLHHHHHHHHHHHHHHLLLL-------L
0020 2auuA   -------LHHH-LLLLLLLLLLEEEEEEELLLLLEEEEELLLLLLEEEEEELLLLLLLLLEEEELLLLLL------------------LLLLLLEEEELL-LLLLLLLLEEEEEEEEEEEEELLEELLEEEEEELLLLLLLLLLLHHHLLHHHHHHHHHHHHHHLLLL--------
0021 1obwA   -------LHHH-LLLLLLLLLLEEEEEEELLLLLEEEEELLLLLLEEEEEELLLLLLLLLEEEELLLLLL------------------LLLLLLEEEELL-LLLLLLLEEEEEEEEEEEEEELLEELLEEEEEELLLLLLLLLLLHHHLLHHHHHHHHHHHHHHLLLL-------L
0022 5c5vA   LLLEEEELHHHHLLLLLL--LLEEEEEEELLLLLLEEELLLLLLLEEELLLLLL-LLLLLEEEELLLLLLLLL-LLLLL------LLLLLLLLLEEEELLLL-LLLLLLEEEEEEEEELEEELLEELLEEEEEELLLLLLLLLLLLHHHLLHHHHHHHHHHHHHLLHH-------L
0023 2prdA   ------LLHHH-LLLLLLLLLEEEEEEEELLLLLEEEEEELLLLEEEEEEELLLLLLLLLEEEELLLLLLL------------------LLLLLEEEEELL-LLLLLLLEEEEEEEEEEEEELLLEEEEEEEEELLLHHHLLLLLHHHLLHHHHHHHHHHHHHHLLHHHHH-----
0024 2au6A   -------LHHH-LLLLLLLLLLEEEEEEELLLLLEEEEELLLLLLEEEEEELLLLLLLLLEEEELLLLLL------------------LLLLLLEEEELL-LLLLLLLLEEEEEEEEEEEEELLEELLEEEEEELLLLLLLLLLLHHHLLHHHHHHHHHHHHHHLLLL-------L
0025 1inoA   -------LLLL-LLLLLLLLLLEEEEEEELLLLLEEEEELLLLLLEEEEEELLLLLLLLLEEEELLLLLL------------------LLLLLLEEEELL-LLLLLLLLEEEEEEEEEEEEELLEELLEEEEEELLLHHHLLLLLLLLLLHHHHHHHHHHHHHHLLLL-------L
0026 5wrtB   -LLLLLLLLLLLLLLLLLL-LLEEEEEEELLLEEELEEELLLLLLEEELLEEE--EEELLEEEELLLLLLLLL--LLLLHHHLL--LEELLLLLEEEELLLLLL-LLLLEEEEEEEEEEEEELLEEEEEEEEEELLLLHHHLLLHHHHLLLHHHHHHHHHHHHHLLHH--------
0027 1mjzA   -------LHHH-LLLLLLLLLLEEEEEEELLLLLEEEEELLLLLLEEEEEELLLLLLLLLEEEELLLLLL------------------LLLLLLEEEELL-LLLLLLLLEEEEEEEEEEEEELLEELLEEEEEELLLLLLLLLLLHHHLLHHHHHHHHHHHHHHLLLL-------L
0028 4um4C   -------LHHH-LLLEEELLLEEEEEEEELLLLLEEEEELLLLLLEEEEEELLLLLLLLLEEEEELLLLL------------------LLLLLLEEEELL-LLLLLLLLEEEEEEEEEEEEELLLLLLEEEEEELLLLLLLLLLLHHHLLHHHHHHHHHHHHHHLLLL-------L
0029 2au9A   ------LHHH--LLLLLLLLLLEEEEEEELLLLLEEEEELLLLLLEEEEEELLLLLLLLLEEEELLLLLL------------------LLLLLLEEEELL-LLLLLLLLEEEEEEEEEEEEELLEELLEEEEEELLLLLLLLLLLHHHLLHHHHHHHHHHHHHHLLLL--------
0030 6mt2D   ------LLHHHHLLLLLLLLLEEEEEEEELLLLLEEEEELLLLLLEEEEEELLLLLLLLLEEEELLLLLL------------------LLLLLLEEEEELL-LLLLLLLEEEEEEEEEELLLLLLLLLEEEEEELLLLLLLLLLLHHHLLHHHHHHHHHHHHHHHHH---------
0031 5wrtA   -LLLLEELLLLLLLLLLLL-LLEEEEEEELLLEEELEEELLLLLLEEELLEEE--EEELLEEEELLLLLLLL---LLLLHHHLL--LEELLLLLEEEELLLLLL-LLLLEEEEEEEEEEEEELLEEEEEEEEEELLLL-LLHHLLLHHHHLLHHHHHHHHHHHHLLHH--------
0032 1mjwA   -------LHHH-LLLLLLLLLLEEEEEEELLLLLEEEEELLLLLLEEEEEELLLLLLLLLEEEELLLLLL------------------LLLLLLEEEELL-LLLLLLLLEEEEEEEEEEEEELLEELLEEEEEELLLLLLLLLLLHHHLLHHHHHHHHHHHHHHLLLL-------L
0033 5cuyA   LLLEEEELHHHHLLLLLL--LLEEEEEEELLLLLLEEELLLLLLLEEELLLLLL-LLLLLEEEELLLLLLLLL--LLLL-----LLLEELLLLLEEEELLLL-LLLLLLEEEEEEEEEEEEELLLEEEEEEEEELLLLLHHHLLLHHHHHLLHHHHHHHHHHHHLLHHHLL-----
0034 6n1cA   ----------LLLLLLLLLLLLEEEEEEELLLLLEEEEELLLLLLEEEEEELLLLLLLLLEEEEELLLLLL------------------LLLLEEEEELLLLLL-LLLLEEEEEEEEEEEEELLEELLEEEEEELLLHHHLLLLLHHHLLHHHHHHHHHHHHHHLLLL-------L
0035 5h4fA   -------LLLL-LLLLLLLLLLEEEEEEELLLLLEEEEELLLLLLEEEEEELLLLLLLLLEEEELLLLLL------------------LLLLLLEEEELL-LLLLLLLLEEEEEEEEEEEEELLLEEEEEEEEELLLLLLLLLLLHHHLLHHHHHHHHHHHHHHLLLL-------L
0036 5teaD   -------LHHHHLLLEE-LLLEEEEEEEELLLLLEEEEEELLLLEEEEEEELLHHHLLLLEEEELLLLLL------------------LLLLLLEEEELLL-LLLLLLLEEEEEEEEEEEEELLEELLEEEEEELLLLLLLLLLLHHHLLHHHHHHHHHHHHHHLLLLL-------
0037 6n1cE   ----------LLLLLLLLLLLLEEEEEEELLLLLEEEEELLLLLLEEEEEELLLLLLLLLEEEEELLLLLL------------------LLLLEEEEELLLLLL-LLLLEEEEEEEEEEEEELLEEEEEEEEEELLLHHHLLLLLHHHLLHHHHHHHHHHHHHHLLLL-------L
0038 1mjwB   -------LHHH-LLLLLLLLLLEEEEEEELLLLLEEEEELLLLLLEEEEEELLLLLLLLLEEEELLLLLL------------------LLLLLLEEEELL-LLLLLLLLEEEEEEEEEEEEELLEEEEEEEEEELLLLLLLLLLLHHHLLHHHHHHHHHHHHHHLLLL-------L
0039 6k27C   ---------HHHLLLLLLLLLLEEEEEEELLLLLEEEEELLLLLLEEEEEELLLLLLLLLEEEELLLLLL------------------LLLLLLEEEELLL-LLLLLLLEEEEEEEEEEEEELLEELLEEEEEELLLLLLLLLLLHHHLLHHHHHHHHHHHHHHLLLL-------L
0040 1udeB   ----------LLLLLLLLLLLLEEEEEEELLLLLEEEEELLLLLLEEEEEELLLLLLLLLEEEELLLLLLLL------------------LLLLEEEELL-LLLLLLLEEEEEEEEEEEEEELLLLLLEEEEEELLLHHHLLLLLHHHLLHHHHHHHHHHHHHHLLHH--------
0041 3eiyA   -------LHHH-LLLLLLLLLLEEEEEEELLLLLEEEEEELLLLEEEEEEELLLLLLLLLEEEEELLLLL------------------LLLLLEEEEELLLLLL-LLLLEEEEEEEEEEEEELLEEEEEEEEEELLLHHHLLLLLHHHLLHHHHHHHHHHHHHHLLLL-------L
0042 3ld3A   -----------LLLLLLLLLLLEEEEEEELLLLLEEEEELLLLLLEEEEEELLLLLLLLLEEEELLLLLL------------------LLLLLLEEEELL-LLLLLLLLEEEEEEEEEEEEELLEELLEEEEEELLLHHHLLLLLHHHLLHHHHHHHHHHHHHHLLLL-------L
0043 3d53D   --------LHHHLLLLLLL-LLEEEEEEELLLLLEEEEELLLLLLEEEEEELLLLLLLLLEEEELLLLLLL------------------LLLLLEEEELLL-LLLLLLLEEEEEEEEEEEEELLEEEEEEEEEELLLHHHLLLLLHHHLLHHHHHHHHHHHHHHLLLL-------L
0044 5cuvB   LLLEEELLLLLLLLLLLL--LLEEEEEEELLLLLLEEELLLLLLLEEELLLLLL-LLLLLEEEELLLLLLLLL--LEEL-----LLEELLLLLLEEEELLLL-LLLLLLEEEEEEEEEEEEELLEEEEEEEEEELLLLLHHHLLLHHHHHLLHHHHHHHHHHHHLLHHH------L
0045 4z70C   -------------------LLLEEEEEEELLLLLEEEEELLLLLLEEEEEELLLLLLLLLEEEEELLLLL------------------LLLLLLEEEELL-LLLLLLLEEEEEEEEEEEEEELLEELLEEEEEELLLHHHLLLLLHHHLLHHHHHHHHHHHHHHLLLL-------L
0046 3emjA   ------------LLLLLLL-LLEEEEEEELLLLLEEEEELLLLLLEEEEEELLLLLLLLLEEEELLLLLLL------------------LLLLLEEEELLL-LLLLLLLEEEEEEEEEEEEELLEEEEEEEEEELLLHHHLLLLLHHHLLHHHHHHHHHHHHHHLLLL-------L
```

PDBe Fold v2.59. (src3) 14 Apr 2014 result file.

RESULT SUMMARY

##	Q-score	P-score	Z-score	RMSD	Nalgn	Nsse	Ngaps	Seq-%	Nmd	Nres-Q	Nsse-Q	Nres-T	Nsse-T	Query	Target
1	1	41.41	19.31	0.000	206	10	0	1	0	206	10	206	10	PDB 6we5:A	PDB 6we5:A
2	0.9867	29.22	16.16	0.349	206	10	0	1	0	206	10	206	10	PDB 6we5:A	PDB 6we5:B
3	0.9789	25.05	14.94	0.325	205	10	0	1	0	206	10	206	10	PDB 6we5:A	PDB 6we5:C
4	0.6942	13.32	11.12	1.179	169	7	4	0.3669	0	206	10	173	9	PDB 6we5:A	PDB 5ty5:F
5	0.6902	12.96	10.98	1.179	169	7	4	0.3669	0	206	10	174	9	PDB 6we5:A	PDB 5ty5:D
6	0.6801	12.08	10.49	1.192	168	7	6	0.369	0	206	10	174	9	PDB 6we5:A	PDB 3r5v:F
7	0.6795	12.73	10.77	1.196	168	7	6	0.369	0	206	10	174	9	PDB 6we5:A	PDB 3r5v:E
8	0.6777	12.81	10.8	1.207	168	7	6	0.369	0	206	10	174	9	PDB 6we5:A	PDB 3r5v:D
9	0.6771	12.88	10.83	1.211	168	7	6	0.369	0	206	10	174	9	PDB 6we5:A	PDB 5ty5:E
10	0.6768	12.77	10.79	1.188	168	7	6	0.369	0	206	10	175	9	PDB 6we5:A	PDB 3q3l:F
11	0.6768	12.55	10.69	1.188	168	7	6	0.369	0	206	10	175	9	PDB 6we5:A	PDB 3i98:F
12	0.6748	11.93	10.43	1.227	169	7	5	0.3609	0	206	10	176	9	PDB 6we5:A	PDB 3q5v:B
13	0.6747	12.77	10.79	1.174	167	7	6	0.3713	0	206	10	174	9	PDB 6we5:A	PDB 3q3l:E
14	0.6746	12.51	10.68	1.175	167	7	6	0.3713	0	206	10	174	9	PDB 6we5:A	PDB 3i98:E
15	0.6705	12.08	10.49	1.228	168	7	6	0.369	0	206	10	175	9	PDB 6we5:A	PDB 3q3l:D
16	0.6689	11.93	10.43	1.238	168	7	6	0.369	0	206	10	175	9	PDB 6we5:A	PDB 3i98:D
17	0.6596	11.55	10.31	1.247	167	7	6	0.3713	0	206	10	175	10	PDB 6we5:A	PDB 3r6e:C
18	0.6547	12.66	10.74	1.225	165	7	7	0.3697	0	206	10	173	9	PDB 6we5:A	PDB 3r6e:D
19	0.6493	12.11	10.55	1.361	168	7	5	0.3512	0	206	10	175	10	PDB 6we5:A	PDB 3r6e:F
20	0.6178	14.32	11.27	1.315	162	8	8	0.2901	0	206	10	173	9	PDB 6we5:A	PDB 6k27:F
21	0.6168	14.39	11.39	1.321	162	8	8	0.2901	0	206	10	173	10	PDB 6we5:A	PDB 6k27:G
22	0.6166	12.01	10.46	1.322	162	7	8	0.2901	0	206	10	173	8	PDB 6we5:A	PDB 6k27:C
23	0.6152	14.2	11.22	1.331	162	8	8	0.2901	0	206	10	173	9	PDB 6we5:A	PDB 6k27:D
24	0.6151	14.36	11.29	1.332	162	8	8	0.2901	0	206	10	173	9	PDB 6we5:A	PDB 6k27:H
25	0.6141	16.35	12.06	1.232	159	8	7	0.3082	0	206	10	171	9	PDB 6we5:A	PDB 6n1c:D
26	0.6127	14.53	11.35	1.298	161	8	7	0.2919	0	206	10	173	9	PDB 6we5:A	PDB 6k27:B
27	0.6098	14.24	11.24	1.367	162	8	8	0.2901	0	206	10	173	9	PDB 6we5:A	PDB 6k27:E
28	0.6087	14.07	11.17	1.374	162	8	8	0.2901	0	206	10	173	9	PDB 6we5:A	PDB 6k27:A
29	0.6064	13.9	11.56	1.440	164	8	5	0.311	0	206	10	175	11	PDB 6we5:A	PDB 5h4f:A
30	0.6055	11.48	10.23	1.395	162	7	8	0.2901	0	206	10	173	8	PDB 6we5:A	PDB 6ki8:B
31	0.6053	13.75	11.04	1.396	162	8	8	0.284	0	206	10	173	9	PDB 6we5:A	PDB 6ki7:B
32	0.6052	13.95	11.12	1.397	162	8	8	0.284	0	206	10	173	9	PDB 6we5:A	PDB 6ki7:C
33	0.6016	13.43	10.91	1.467	163	8	7	0.2883	0	206	10	173	9	PDB 6we5:A	PDB 6k21:A
34	0.5991	15.98	11.92	1.230	157	8	8	0.3121	0	206	10	171	9	PDB 6we5:A	PDB 6n1c:B
35	0.598	13.77	11.5	1.463	161	8	7	0.354	0	206	10	170	11	PDB 6we5:A	PDB 2bqy:A
36	0.5972	13.66	11.15	1.235	155	7	7	0.3097	0	206	10	167	8	PDB 6we5:A	PDB 6n1c:C
37	0.5948	12.39	10.74	1.364	159	7	8	0.3145	0	206	10	171	8	PDB 6we5:A	PDB 6n1c:F
38	0.5942	12.11	10.5	1.321	159	7	8	0.2956	0	206	10	173	8	PDB 6we5:A	PDB 6ki8:C
39	0.5938	12.86	11.14	1.492	162	8	6	0.2901	0	206	10	172	11	PDB 6we5:A	PDB 3sw5:B
40	0.5937	12.94	11.17	1.492	162	8	6	0.2901	0	206	10	172	11	PDB 6we5:A	PDB 3sw5:E
41	0.593	12.53	10.73	1.475	162	8	6	0.284	0	206	10	173	9	PDB 6we5:A	PDB 6ki7:F
42	0.593	14.25	11.69	1.402	160	8	6	0.3125	0	206	10	172	11	PDB 6we5:A	PDB 5h4f:B
43	0.5925	11.69	10.49	1.498	161	8	6	0.3168	0	206	10	170	10	PDB 6we5:A	PDB 3tr4:F
44	0.5912	14.52	11.44	1.391	160	8	8	0.2875	0	206	10	173	10	PDB 6we5:A	PDB 6ki7:A
45	0.5902	11.89	10.25	1.446	161	8	9	0.2857	0	206	10	173	10	PDB 6we5:A	PDB 5tea:E
46	0.59	11.14	10.08	1.447	161	7	8	0.2857	0	206	10	173	8	PDB 6we5:A	PDB 6ki8:A
47	0.5898	13.21	10.91	1.401	160	8	8	0.2875	0	206	10	173	10	PDB 6we5:A	PDB 6ki7:E
48	0.5883	12.94	11.17	1.380	158	8	9	0.3101	0	206	10	170	11	PDB 6we5:A	PDB 3i4q:A
49	0.5875	13.89	11.19	1.415	160	8	7	0.2875	0	206	10	173	10	PDB 6we5:A	PDB 6ki7:G
50	0.5874	12.3	10.64	1.417	160	8	8	0.2875	0	206	10	173	9	PDB 6we5:A	PDB 6ki7:H

51	0.583	11.34	10.5	1.458	157	7	6	0.3631	0	206	10	166	10	PDB 6we5:A	PDB 2bqx:A
52	0.5827	11.04	10.15	1.438	157	7	6	0.293	0	206	10	167	8	PDB 6we5:A	PDB 3d53:E
53	0.5826	15.93	11.9	1.303	158	8	9	0.3101	0	206	10	175	9	PDB 6we5:A	PDB 6n1c:E
54	0.5818	11.4	10.36	1.516	159	8	6	0.3208	0	206	10	168	10	PDB 6we5:A	PDB 3tr4:D
55	0.5815	11.62	10.46	1.499	160	8	6	0.3187	0	206	10	171	10	PDB 6we5:A	PDB 3tr4:E
56	0.5812	12.43	10.75	1.196	149	7	5	0.3154	0	206	10	160	8	PDB 6we5:A	PDB 4z70:C
57	0.5805	13.28	11.15	1.379	156	8	8	0.2885	0	206	10	168	10	PDB 6we5:A	PDB 3emj:C
58	0.5763	13.66	11.15	1.272	157	7	9	0.3057	0	206	10	176	8	PDB 6we5:A	PDB 6n1c:A
59	0.5734	11.05	9.871	1.555	161	7	6	0.2857	0	206	10	173	7	PDB 6we5:A	PDB 6ki7:D
60	0.5729	11.56	10.38	1.313	150	7	4	0.3133	0	206	10	160	8	PDB 6we5:A	PDB 4z71:C
61	0.5705	11.4	10.36	1.443	155	8	7	0.2903	0	206	10	166	10	PDB 6we5:A	PDB 3emj:B
62	0.5702	11.81	10.49	1.277	149	7	5	0.3154	0	206	10	160	8	PDB 6we5:A	PDB 4z74:C
63	0.5702	12.92	11.01	1.446	155	8	7	0.2903	0	206	10	166	10	PDB 6we5:A	PDB 3emj:G
64	0.5598	11.35	10.29	1.352	149	7	5	0.3154	0	206	10	160	8	PDB 6we5:A	PDB 4z73:C
65	0.5596	11.7	10.44	1.304	149	7	5	0.3154	0	206	10	162	8	PDB 6we5:A	PDB 4z70:B
66	0.5583	11.42	10.14	1.390	155	8	8	0.3613	0	206	10	172	10	PDB 6we5:A	PDB 1ygz:F
67	0.5577	12.71	10.7	1.393	155	8	8	0.3613	0	206	10	172	10	PDB 6we5:A	PDB 1ygz:E
68	0.5577	11.39	10.13	1.394	155	8	8	0.3613	0	206	10	172	10	PDB 6we5:A	PDB 1ygz:D
69	0.5553	11.99	10.57	1.331	148	7	5	0.3041	0	206	10	160	8	PDB 6we5:A	PDB 4z73:J
70	0.5497	9.858	9.489	1.371	148	7	5	0.3108	0	206	10	160	7	PDB 6we5:A	PDB 4z73:H
71	0.546	6.457	8.183	1.916	166	8	5	0.2711	0	206	10	174	11	PDB 6we5:A	PDB 3fq3:I
72	0.5427	10.41	9.871	1.422	148	7	5	0.3108	0	206	10	160	8	PDB 6we5:A	PDB 4z74:H
73	0.5038	6.726	8.163	1.936	159	7	9	0.2704	0	206	10	172	10	PDB 6we5:A	PDB 4xel:A
